# Exploring the Causal Link Between Plasma Lipidome and Trigeminal Neuralgia Using Bidirectional Mendelian Randomization

**DOI:** 10.1155/prm/8746245

**Published:** 2025-07-25

**Authors:** Yuhang Peng, Xiaolin Zhang, Jinhua Guo, Mingxin Chen, Yuan Cheng, Jianhe Yue, Yongxiang Jiang

**Affiliations:** ^1^Department of Neurosurgery, The Second Affiliated Hospital of Chongqing Medical University, Chongqing, China; ^2^Department of Osteology, The Second Affiliated Hospital of Chongqing Medical University, Chongqing, China

**Keywords:** causally association, Mendelian randomization, plasma lipidome, trigeminal neuralgia

## Abstract

**Background:** Trigeminal neuralgia (TN) is a prevalent neurological disorder characterized by recurrent acute pain localized within the distribution area of the trigeminal nerve. This condition places a severe psychological and emotional burden on patients. Although lipids are associated with many diseases, their relationship with TN remains unclear. This study aims to investigate the causal association between plasma lipidome and TN using a bidirectional two-sample Mendelian randomization (MR) approach, with the ultimate goal of informing potential therapeutic strategies for TN management.

**Methods:** We conducted a bidirectional two-sample MR analysis to systematically assess the causal relationship between plasma lipidome and TN. Genome-wide association study (GWAS) summary statistics for plasma lipidome and TN were obtained from publicly available datasets. The primary causal inference was performed using inverse variance weighted (IVW) regression, with complementary analyses including MR-Egger regression, weighted mode, simple mode, weighted median, and MR pleiotropy residuals and outliers (MR-PRESSO) to test for and adjust potential pleiotropy. Comprehensive sensitivity analyses were implemented to verify the robustness of our findings, including heterogeneity testing, leave-one-out analysis, and examination of directional pleiotropy. This multianalytical approach provides a rigorous framework for elucidating the potential role of plasma lipidome dysregulation in TN pathogenesis.

**Results:** Our forward MR analysis results demonstrated that genetically predicted glycerophospholipids (GP) and glycerolipid family (GL) exert significant causal effects on TN risk. More specifically, phosphatidylinositol (PI) in the GP, as well as diacylglycerol and triacylglycerol in the GL, were significantly associated with reduced TN risk (*p* < 0.05, OR < 1). However, distinct molecular configurations of phosphatidylcholine (PC) and phosphatidylethanolamine (PE) within the GP class exhibited differential impacts on TN susceptibility. The reverse MR analysis identified eight configurations of PC reduced TN risk (*p* < 0.05, OR < 1), with PC (18:0_18:2) showing a particularly notable bidirectional causal relationship with TN. Rigorous sensitivity analyses confirmed the absence of both heterogeneity (Cochran's *Qp* > 0.05) and horizontal pleiotropy (MR-Egger intercept *p* > 0.05) across all examined lipid species, supporting the robustness of these findings.

**Conclusions:** This MR study establishes causal links between specific plasma lipidomes and TN risk, identifying protective lipid species and revealing a bidirectional relationship for PC, offering potential therapeutic targets for TN management.

## 1. Introduction

TN is a chronic neurological disorder characterized by sudden, intense, brief episodes of sharp, unilateral facial pain along the trigeminal nerve distribution [1, 2]. Everyday actions like eating, talking, or even brushing teeth can trigger these painful episodes [3]. It affects about 12.6–27.0 people per 100,000 each year, with women being more likely to develop it than men (Female: Male ratio ≈ 3:2) [4]. The intense, unpredictable pain often leads to anxiety, depression, and sleep problems, greatly reducing patients' quality of life [5]. While treatments like pain medications and surgery can help manage symptoms, they do not address the root cause of TN [6]. This gap highlights the need for research into the underlying mechanisms of TN to develop better treatments.

Lipids play pivotal roles in human physiological homeostasis, with accumulating evidence demonstrating their pathophysiological associations with diverse clinical diseases, like atrial fibrillation [7], Alzheimer's disease [8], and diabetes mellitus [9]. The rapid expansion of GWAS in lipidomics [10] and TN [11] has created novel methodological paradigms for investigating potential causal relationships between lipid metabolism dysregulation and TN development.

Mendelian randomization (MR) represents an advanced genetic epidemiological method that leverages genetic variants as instrumental variables (IVs) to infer causal relationships. By leveraging the random assortment of alleles during meiosis, MR minimizes confounding biases and avoids reverse causation—limitations that frequently compromise observational studies. This methodology provides a robust alternative to randomized controlled trials (RCTs) when clinical trials are ethically or practically unfeasible [12, 13]. In the present study, we employ a bidirectional two-sample MR design to systematically evaluate the causal association between plasma lipidome and TN. Our investigation aims to not only elucidate potential pathological mechanisms but also identify novel targets for both prevention and therapeutic intervention.

## 2. Materials and Methods

### 2.1. Study Design and Data Source

**Figure 1:**
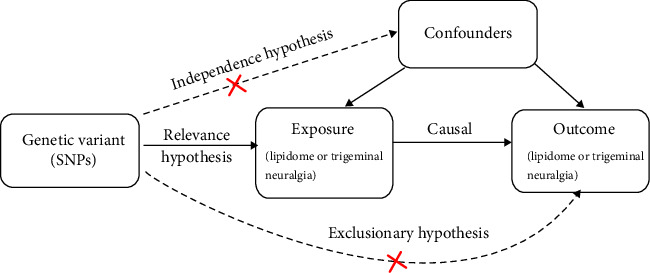
The three major assumptions of Mendelian randomization.

This study employed a univariate MR approach utilizing pooled GWAS summary statistics to investigate causal relationships between plasma lipid traits and TN. The MR analysis was conducted in strict accordance with three core MR assumptions: (I) Relevance hypothesis: Genetic variations must strongly correlate with the exposure. (II) Independence hypothesis: Genetic variations should be independent of confounding variables. (III) Exclusion hypothesis: Genetic variations must influence the outcome exclusively through the exposure of interest [14] (as shown in Figure 1).

We utilized pooled GWAS summary statistics for lipids traits from the GWAS catalog, comprising comprehensive data on 13 lipid classes derived from 7174 Finnish individuals. The analyzed lipid profiles encompassed four major biochemical categories: Glycerophospholipids (GP), glycerolipids (GL), sphingolipids (SL), and sterols (ST) [15].

TN case-control data were sourced from the FinnGen database (Finngen_R11_G6_TRINEU), comprising aggregated genetic data from European-ancestry populations. The analysis included 800 TN cases and 195,047 controls (total *N* = 195,847).

### 2.2. Selection of IVs

First, by screening the GWAS data, based on the genome-wide significance threshold, the included relevant Single Nucleotide Polymorphisms (SNPs) met *p* < 1 ∗ 10^−5^ [16]. In reverse MR, because fewer TN phenotypic SNPs were screened at *p* < 1 ∗ 10^−5^, we used a loose significance threshold (*p* < 5 ∗ 10^−5^) for selection. In addition, to mitigate linkage disequilibrium (LD) induced bias, we performed LD clumping using PLINK's positional-clumping algorithm (window size = 10,000 kb, *r*^2^ threshold = 0.001), retaining the most significant SNPs per locus [17]. Potential pleiotropic confounders were systematically addressed by excluding SNPs associated with established TN risk factors (such as body mass index and C-reactive protein) through PhenoScanner v2.0 queries. Finally, in order to reduce the influence of weak IVs on the included SNPs, the F-statistic was adopted (the calculation formula is *F* = *β*^2^/SE^2^, *β* is the equipotential effect value and SE is the standard error). If the F-statistic of the SNPs is less than 10, it indicates that the SNPs may have a weak IV bias, and this bias should be excluded to avoid affecting the results. Therefore, SNPs with an F value greater than 10 were retained. The SNPS obtained by this multilayer quality control are used as IVs (the specific data of IVs can be found in Supporting Information) for bidirectional MR analysis.

### 2.3. Statistical Analysis

MR analysis of the final IVs was performed using five widely used methods: IVW, MR-Egger regression, weighted median, weighted model and simple model analysis. IVW results are mainly analyzed, because this method usually has high statistical power under the hypothesis of IV effects, and is widely used as the core method of MR Analysis. We used IVW to assess heterogeneity, and the results of the heterogeneity test were represented by the Cochrane *Q* statistic and Q_*p* value. The MR-Egger intercept is used to detect and interpret pleiotropy, while abnormal SNPS and level pleiotropy that may lead to bias are identified by MR pleiotropy residuals and outliers (MR-PRESSO) [17]. To further assess the stability of the findings, a leave-one-out sensitivity analysis was performed. This approach evaluated the impact of individual SNPs on the overall results by sequentially excluding each SNP and analyzing the aggregated effect of the remaining SNPs [18, 19]. The results were expressed as odds ratios (OR) with corresponding 95% confidence intervals (95% CI). A *p* value of less than 0.05 was considered statistically significant. All statistical analyses were performed using the Two-Sample MR package in R version 4.4.1.

## 3. Results

### 3.1. Causal Effects of Plasma Lipidome on TN

**Figure 2:**
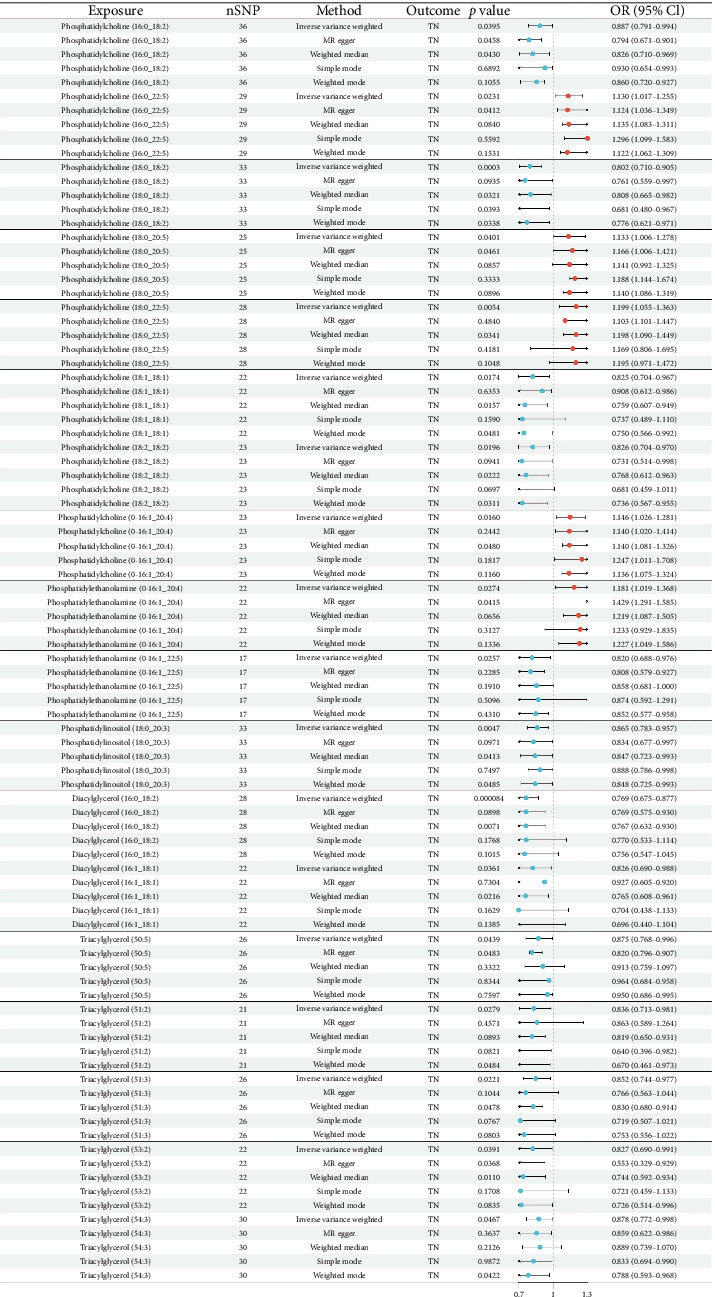
Results of five methods of forward Mendelian randomization.

The results of MR of TN by plasma lipidome are shown in Figure 2. Results showed that diacylglycerol, triacylglycerol, phosphatidylcholine (PC), phosphatidylethanolamine (PE), and phosphatidylinositol (PI) had an effect on TN risk (Figure 2). However, the study also found that different levels of lipids have different effects on TN risk. Details are as follows:

Risk-increasing lipids:
  I. PC (16:0_22:5; OR = 1.130, 95% CI: 1.017–1.255; *p*=0.0231), PC (18:0_20:5; OR = 1.133, 95% CI: 1.006–1.278; *p*=0.0401), PC (18:0_22:5; OR = 1.199, 95% CI: 1.055–1.363, *p*=0.0054), PC (O-16:1/_20:4; OR = 1.146, 95% CI: 1.026–1.281, *p*=0.0257)
  II. PE (O-16:1_20:4; OR = 1.81, 95% CI: 1.019–1.368, *p*=0.0274).
Protective lipids:
  I. PC (16:0_18:2; OR = 0.887, 95% CI: 0.791–0.994, *p*=0.0395), PC (18:0_18:2; OR = 0.802, 95% CI: 0.710–0.905, *p*=0.0003), PC (18:1_18; 1; OR = 0.825, 95% CI: 0.704–0.967, *p*=0.0174), PC (18:2_18:2; OR = 0.826, 95% CI: 0.704–0.970, *p*=0.0160), PE (O-16:1:22:5; OR = 0.820, 95% CI: 0.688–0.976, *p*=0.0257), PI (18:0_20:3; OR = 0.865, 95% CI: 0.783–0.957, *p*=0.0047),
  II. PE (O-16:1:22:5; OR = 0.820, 95% CI: 0.688–0.976, *p*=0.0257),
  III. PI (18:0_20:3; OR = 0.865, 95% CI: 0.783–0.957, *p*=0.0047).
  IV. Diacylglycerol (16:0_18:2; OR = 0.769, 95% CI: 0.675–0.877, *p*=0.000084), diacylglycerol (16:1_18:1; OR = 0.826, 95% CI: 0.690–0.988, *p*=0.0361), triacylglycerol (50:5; OR = 0.875, 95% CI: 0.768–0.996, *p*=0.0439), triacylglycerol (51:2; OR = 0.836, 95% CI: 0.713–0.981, *p*=0.0279), triacylglycerol (51:3; OR = 0.852, 95% CI: 0.744–0.977, *p*=0.0221), triacylglycerol (53:2; OR = 0.827, 95% CI: 0.690–0.991; *p*=0.0391), and triacylglycerol (54:3; OR = 0.878, 95% CI: 0.772–0.998, *p*=0.0467)

Notably, the directionality of effects was consistent across all analytical methods, with IVW showing the strongest statistical power. The observed effects of different levels of PC on TN are particularly distinct, indicating that there is a potential structure-activity relationship in plasma lipids in the pathogenesis of TN.

### 3.2. Forward Sensitivity Analysis

**Figure 3:**
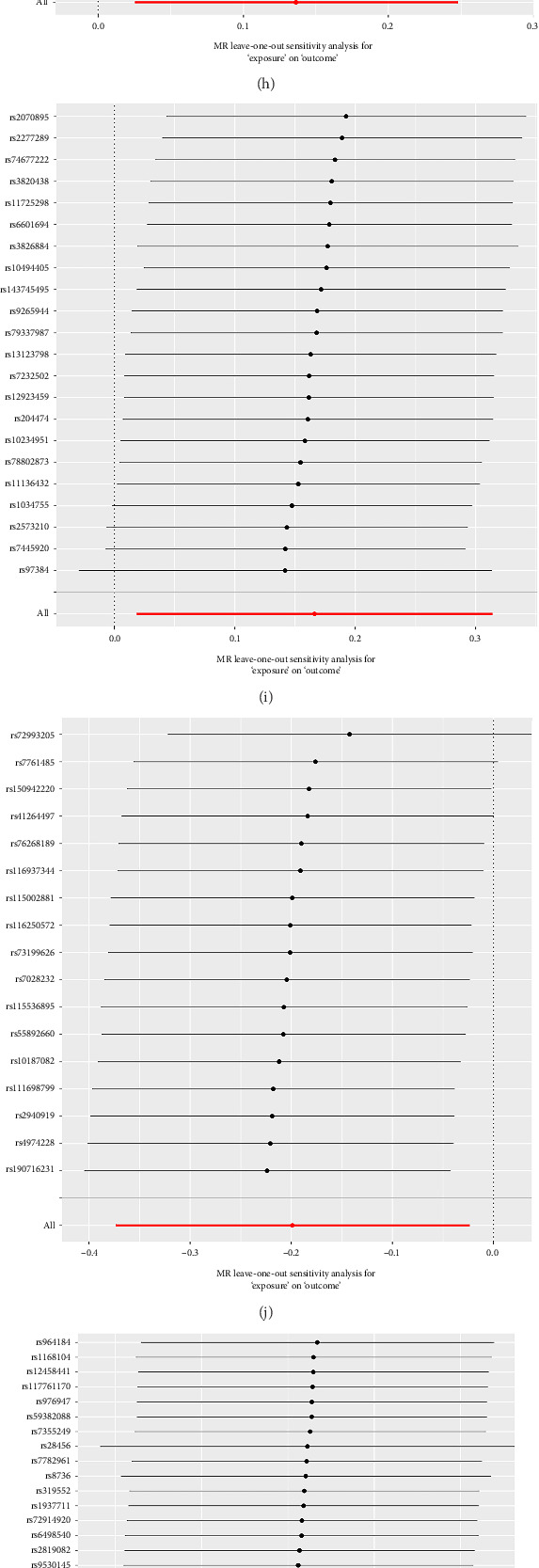
The leave one out plot of forward MR analyses. (a) Phosphatidylcholine (16:0_18:2); (b) phosphatidylcholine (16:0_22:5); (c) phosphatidylcholine (18:0_18:2); (d) phosphatidylcholine (18:0_20:5); (e) phosphatidylcholine (18:0_22:5); (f) phosphatidylcholine (18:1_18:1); (g) phosphatidylcholine (18:2_18:2); (h) phosphatidylcholine (O-16:1_20:4); (i) phosphatidylethanolamine (O-16:1_20:4); (j) phosphatidylethanolamine (O-16:1_22:5); (k) phosphatidylinositol (18:0_20:3); (l) diacylglycerol (16:0_18:2); (m) diacylglycerol (16:1_18:1); (n) triacylglycerol (50:5); (o) triacylglycerol (51:2); (p) triacylglycerol (51:3); (q) triacylglycerol (53:2); (r) triacylglycerol (54:3).

The results of sensitivity analysis showed that the phenotypes of the 18 lipid levels used for MR analysis of TN were not heterogeneous (Q-*p* value > 0.05), nor were there horizontal pleiotropy (MR-Egger's intercept method *p* > 0.05), which proved that the result of causal robustness was credible (Table 1). Both the leave-one method and the funnel plot show reliable data (Figure 3). Meanwhile, MR-PRESSO analysis showed no outliers and pleiotropy, as shown in Table 1.

### 3.3. Causal Effects of TN on Plasma Lipidome

**Figure 4:**
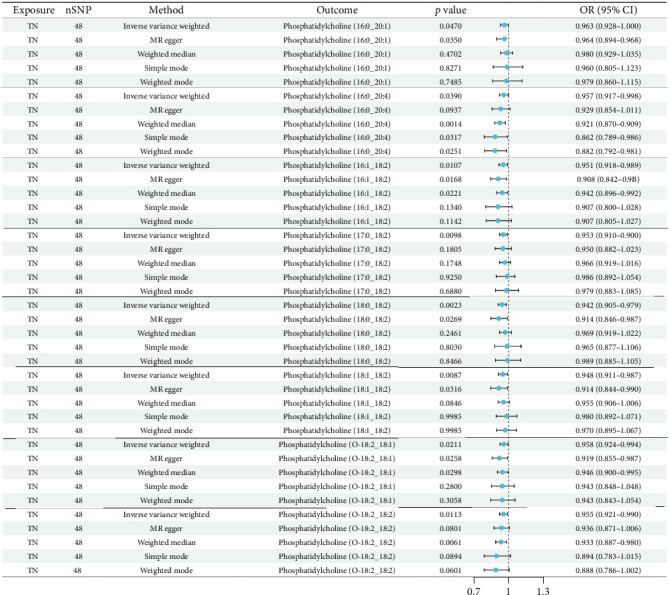
Results of five methods of reverse Mendelian randomization.

The results of MR of plasma lipidome by TN are shown in Figure 4. The results revealed significant causal effects of TN on PC. The main manifestation is that TN can reduce PC in blood lipids. The specific results are as follows: PC (16:0_20:1; OR = 0.963, 95% CI: 0.928–1.000; *p*=0.0470), PC (16:0_20:4; OR = 0.957, 95% CI: 0.917–0.998; *p*=0.0390), PC (16:1_18:2; OR = 0.951, 95% CI: 0.916–0.989; *p*=0.0107), PC (17:0 _18:2; OR = 0.953, 95% CI: 0.918–0.988; *p*=0.0098), PC (18:0_18:2; OR = 0.942, 95% CI: 0.906–0.979; *p*=0.0023), PC (18:1_18:2; OR = 0.948, 95% CI: 0.911–0.987, *p*=0.0087), PC (O-18:2_18:1; OR = 0.958, 95% CI: 0.924–0.994; *p*=0.0211), and PC (O-18:2_18:2; OR = 0.955, 95% CI: 0.921–0.990; *p*=0.0113).

Meanwhile, the results indicated that PC (18:0_18:2) emerged as a key lipid showing significant associations in both forward (TN risk) and reverse (lipid alteration) MR analyses (Table 2), suggesting a potential feedback loop between this lipid species and TN pathogenesis. All supporting MR methods (MR-Egger, weighted median, etc.) showed effect estimates concordant with IVW directionality, supporting the robustness of these findings.

### 3.4. Reverse Sensitivity Analysis

**Figure 5:**
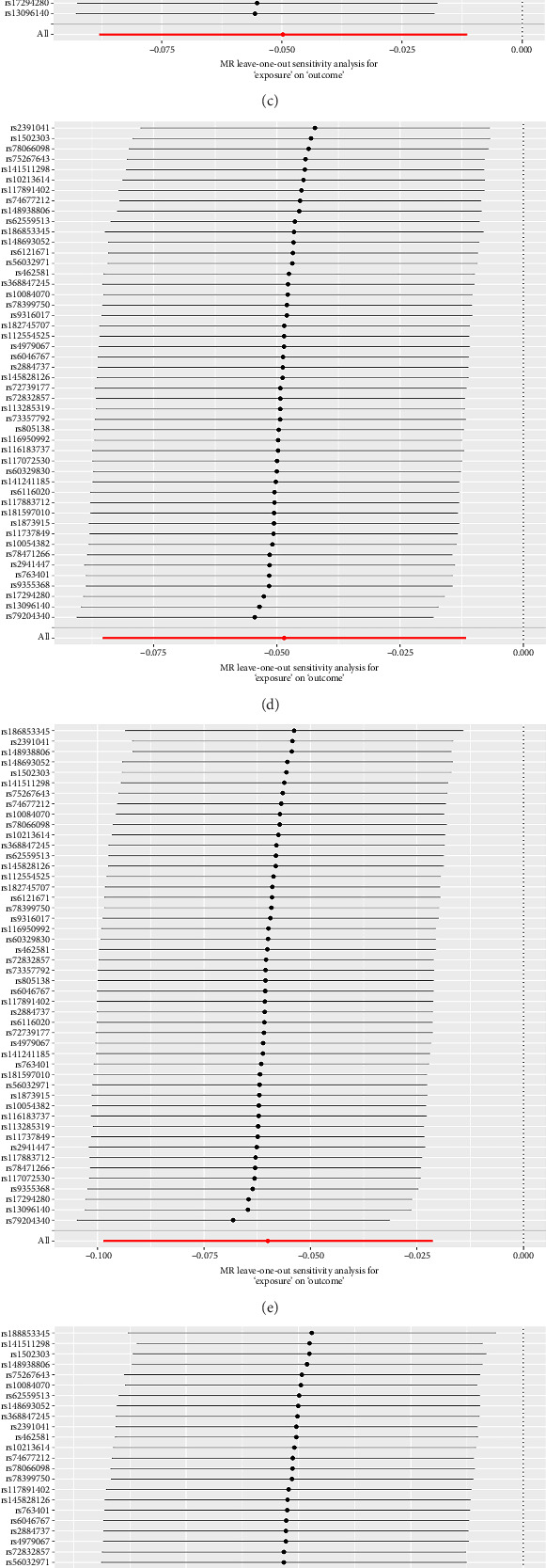
The leave one out plot of reverse MR analyses. (a) Phosphatidylcholine (16:0_20:1); (b) Phosphatidylcholine (16:0_20:4); (c) phosphatidylcholine (16:1_18:2); (d) phosphatidylcholine (17:0_18:2); (e) Phosphatidylcholine (18:0_18:2); (f) phosphatidylcholine (18:1_18:2); (g) phosphatidylcholine (O-18:2_18:1); (h) phosphatidylcholine (O-18:2_18:2).

Sensitivity analysis results showed that there was no heterogeneity in the phenotypes of the above-mentioned 9 lipid levels used for MR analysis of TN (Q-*p* value > 0.05) and no horizontal pleiotropy (MR-Egger's intercept method *p* > 0.05), which proved that the result of causal robustness was credible (Table 3). Both the leave-one method and the funnel plot show reliable data (Figure 5). Meanwhile, MR-PRESSO analysis showed no outliers and pleiotropy, as shown in Table 3.

## 4. Discussion

Our study represents the first comprehensive investigation into the complex causal relationships between plasma lipidome and TN. The results show that diacylglycerol, triacylglycerol, PC, PE, and PI have a certain relationship with the occurrence of TN. Meanwhile, we also found that different lipids have different effects on the risk of TN, and different fatty acid compositions of the same lipids also have different effects on the risk of TN. Through rigorous sensitivity analyses employing MR-Egger regression and weighted median methods, we established the absence of substantial heterogeneity and detected no evidence of horizontal pleiotropy. Therefore, we believe that my research results are relatively reliable.

In our analysis, we primarily relied on IVW as our main analytical approach, given its established statistical power under valid IV assumptions and its widespread adoption as the gold standard in MR Analysis. The results of the sensitivity analysis showed that the lipids used for MR analysis had no heterogeneity (Q-*p* value > 0.05), nor was there horizontal pleiotropy (Mr-Egger's intercept method *p* > 0.05). While some supporting methods (MR-Egger regression, weighted median, etc.) showed nonsignificant *p* values (*p* > 0.05) in Figures 2, 4, their effect estimates consistently aligned in direction with the IVW results. Importantly, as supported by Chen et al. [20], when the IVW method yields significant results (*p* < 0.05) in the absence of pleiotropy and heterogeneity as confirmed by our sensitivity analyses—it provides reliable causal estimates even if other methods are nonsignificant [21, 22]. This methodological consistency, combined with the concordant directional effects across approaches, reinforces the validity of our findings.

In this study, our MR analyses revealed distinct risk profiles for TN associated with different levels of PC and PE. Given the paucity of prior evidence regarding different levels of plasma lipids effects on TN, we hypothesized that different levels of the same type of lipids may play different roles in the inflammatory process involved in TN. Notably, four levels of PC (16:0_22:5, 18:0_20:5, 18:0_22:5, and O-16:1_20:4) exhibited inverse correlations with TN risk, potentially through modulating the biosynthesis of pro-inflammatory mediators such as eicosanoids and cytokines [23, 24]. This mechanistic hypothesis aligns with converging evidence implicating neuroinflammation in both TN disease progression and etiopathogenesis [25, 26]. Phosphatidyl ethanolamine is a major phospholipid found in cell membranes, and specific stimulation can induce changes in the composition of phosphatidyl inositol, thereby regulating the phosphoinositol 3-kinase (PI3K) signaling pathway [27]. PI3K is an enzyme involved in the inositol cycle and the production of inositol triphosphate (IP3), an important second-messenger phospholipid that binds to IP3 receptors in the endoplasmic reticulum, releases intracellular calcium stores, and regulates cell proliferation and autophagy [28, 29]. Calcium surges regulated by IP3 can directly or indirectly induce apoptosis [30]. Therefore, different levels of PI may have different effects on TN. These observations warrant further investigation to elucidate the molecular mechanisms at play and explore the clinical implications of these findings.

It is worth noting that our research found that PC (18:0–18:2) had a significant correlation in both forward and reverse MR analyses. The dual fatty acid composition of PC (18:0_18:2) may underlie its pathological relevance: the saturated stearic acid (18:0) confers membrane rigidity, while the polyunsaturated linoleic acid (18:2) serves as a precursor for pro-inflammatory eicosanoids (e.g., prostaglandin E2) capable of sensitizing trigeminal nociceptors [31]. The inverse causality direction (TN on lipid alteration) may reflect chronic stress-induced activation of the hypothalamic-pituitary-adrenal (HPA) axis, which upregulates stearoyl-CoA desaturase-1 (SCD1) and subsequently modifies circulating lipid profiles [32]. These findings collectively advance our mechanistic understanding of lipid-mediated TN pathogenesis and highlight potential therapeutic targets.

Intriguingly, the randomization results of MR also pose some problems and challenges for us. In our traditional understanding, monoglycerides, diglycerides, and triglycerides increase the risk of various diseases [33]. However, our findings suggest that triglycerides may be a protective factor for TN. Like most complex diseases, the common variations found in GWAS can only explain a small part of the total heritability of the disease, while rare variations throughout the genome may also play an important role in the development of the disease. Therefore, it is necessary to combine basic research and clinical research to further clarify the causal relationship [34]. It is worth noting that diglyceride and triglyceride exhibit different structural changes. Previous studies have mainly studied diglyceride and triglyceride as a whole, without specific segmentation based on their structural forms. Therefore, further research is needed to determine which specific diglycerol and triglyceride structural forms may play a preventive role. These findings highlight the complexity of lipid levels in TN etiology and the need for further research to uncover the exact mechanisms involved.

Despite the novel insights provided, this study has several limitations. First, since the traditional *p* < 5 ∗ 10^−8^ cannot screen out enough SNPS, in order to obtain enough SNPS when screening liposomal SNPS, we adopted a more lenient threshold of 5 ∗ 10^−5^. The use of this threshold may have increased the likelihood of potential bias, but a loose threshold significantly improves statistical power and allows us to detect associations with small effect sizes, which is particularly important in exploratory studies, so we adopted this threshold. Secondly, the majority of participants in this study were of European descent, which limits the generalization of our findings to other populations. Additionally, this study aims to explore the causal relationship between plasma lipids and TN outcomes, with its core analysis based on preselected IVs and exposure-outcome pairs. Although multiple comparisons are involved, MR analysis places greater emphasis on evaluating the robustness of results through sensitivity methods (such as MR-Egger and weighted median) and IV assumption tests (such as heterogeneity Q values and pleiotropy intercepts), rather than relying solely on *p* value correction. Furthermore, we hope to identify as many potential therapeutic targets as possible, and in line with the research by Yuan et al. [35], we chose not to perform multiple testing correction.

## 5. Conclusion

This pioneering MR study establishes a causal nexus between plasma lipids and TN pathogenesis, revealing differential risk modulation contingent upon different levels and class of lipid. PC (16:0_22:5; 18:0_22:5; O-16:1_20:4) and PE (O-16:1_20:4) increased the risk of TN. PC (16:0_18:2; 18:0_18:2; 18:1_18; 1; 18:2_18:2), PE (O-16:1:22:5), PI (18:0_20:3), diacylglycerol (16:0_18:2, 16:1_18:1), and triacylglycerol (50:5; 51:2; 51:3; 53:2; 54:3) reduced the risk of TN. PC (18:0_18:2) showing a particularly notable bidirectional causal relationship with TN. The findings implicate lipid metabolism in TN pathogenesis and highlight potential therapeutic targets.

## Figures and Tables

**Table 1:** Forward MR sensitivity analysis.

Exposure	nSNP	Outcome	Cochran's *Q* test		MR-Egger intercept test		MR-PRESSO	
			*Q*	Q-*p* value	Egger_ intercept	*p* value	Outlier	*p* value
Phosphatidylcholine (16:0_18:2)	36	TN	42.37	0.154	0.005	0.780	0	0.226
Phosphatidylcholine (16:0_22:5)	29	TN	20.41	0.813	0.001	0.944	0	0.870
Phosphatidylcholine (18:0_18:2)	33	TN	33.01	0.369	0.007	0.723	0	0.471
Phosphatidylcholine (18:0_20:5)	25	TN	27.93	0.218	−0.006	0.729	0	0.359
Phosphatidylcholine (18:0_22:5)	28	TN	28.88	0.317	0.012	0.499	0	0.431
Phosphatidylcholine (18:1_18:1)	22	TN	23.88	0.248	−0.014	0.607	0	0.305
Phosphatidylcholine (18:2_18:2)	23	TN	27.93	0.142	0.017	0.447	0	0.180
Phosphatidylcholine (O-16:1_20:4)	23	TN	24.06	0.29	0.001	0.956	0	0.429
Phosphatidylethanolamine (O-16:1_20:4)	22	TN	21.22	0.384	−0.001	0.973	0	0.477
Phosphatidylethanolamine (O-16:1_22:5)	17	TN	13.27	0.581	0.003	0.92	0	0.676
Phosphatidylinositol (18:0_20:3)	33	TN	26.62	0.691	0.006	0.695	0	0.766
Diacylglycerol (16:0_18:2)	28	TN	22.8	0.644	0.000043	0.998	0	0.715
Diacylglycerol (16:1_18:1)	22	TN	27.53	0.121	−0.018	0.563	0	0.176
Triacylglycerol (50:5)	26	TN	26.63	0.375	−0.029	0.167	0	0.402
Triacylglycerol (51:2)	21	TN	18.78	0.471	−0.004	0.864	0	0.559
Triacylglycerol (51:3)	26	TN	20.49	0.668	0.017	0.459	0	0.697
Triacylglycerol (53:2)	22	TN	26.28	0.157	0.05	0.123	0	0.121
Triacylglycerol (54:3)	30	TN	18.99	0.898	0.003	0.886	0	0.936

*Note:* nSNP: The number of SNPs.

**Table 2:** Bidirectional MR: phosphatidylcholine (18:0_18:2) and TN.

Exposure	nSNP	Outcome	*p* value	OR	OR_lci95	OR_uci95
Phosphatidylcholine (18:0_18:2)	33	TN	0.0003	0.802	0.71	0.905
TN	40	Phosphatidylcholine (18:0_18:2)	0.0023	0.942	0.906	0.979

*Note:* OR_lci95: The lower limit of the 95% confidence interval of the OR value. OR_uci95: The upper limit of the 95% confidence interval of the OR value.

**Table 3:** Reverse MR sensitivity analysis.

Exposure	nSNP	Outcome	Cochran's *Q* test		MR-Egger intercept test		MR-PRESSO	
			Q	Q-*p* value	Egger_ intercept	*p*	Outlier	*p* value
TN	48	Phosphatidylcholine (16:0_20:1)	50.35	0.342	−0.00008	0.992	0	0.721
TN	48	Phosphatidylcholine (16:0_20:4)	41.04	0.717	−0.004	0.583	0	0.156
TN	48	Phosphatidylcholine (16:1_18:2)	56.6	0.159	0.012	0.175	0	0.201
TN	48	Phosphatidylcholine (18:0_18:2)	57.82	0.134	0.008	0.381	0	0.891
TN	48	Phosphatidylcholine (18:1_18:2)	61.45	0.077	0.009	0.302	0	0.458
TN	48	Phosphatidylcholine (18:2_18:2)	76.46	0.004	0.008	0.45	0	0.339
TN	48	Phosphatidylcholine (O-18:2_18:1)	44.61	0.572	0.011	0.192	0	0.390
TN	48	Phosphatidylcholine (O-18:2_18:2)	49.61	0.37	0.005	0.543	0	0.322

## Data Availability

The datasets used and/or analyzed during the current study are available from the corresponding author on reasonable request.

## Supporting Information

Supporting Information provides the specific data of IVs.
